# Predictive value of individual serum neurofilament light chain levels in short-term disease activity in relapsing multiple sclerosis

**DOI:** 10.3389/fneur.2024.1354431

**Published:** 2024-02-14

**Authors:** Luis Solís-Tarazona, Lars Lau Raket, Javier Cabello-Murgui, Salma Reddam, Silvia Navarro-Quevedo, Sara Gil-Perotin

**Affiliations:** ^1^Research Group in Immunotherapy and Biomodels for Autoimmunity, Instituto de Investigación Sanitaria La Fe, Valencia, Spain; ^2^Clinical Memory Research Unit, Department of Clinical Sciences, Lund University, Lund, Sweden; ^3^Neurology, Hospital Universitario y Politécnico La Fe, Valencia, Spain; ^4^Multiple Sclerosis Unit, Neurology, Hospital Universitario y Politécnico La Fe, Valencia, Spain; ^5^Consorcio Centro de Investigación Biomédica en Red (CIBER), CB06/05/1131, Instituto de Salud Carlos III, Madrid, Spain

**Keywords:** multiple sclerosis, biomarker, relapse, Z-score, RMS

## Abstract

**Background:**

The assessment of serum neurofilament light chain (sNFL) has emerged as a diagnostic and prognostic tool in monitoring multiple sclerosis (MS). However, the application of periodic measurement in daily practice remains unclear.

**Objective:**

To evaluate the predictive value of individual sNFL levels in determining disease activity in patients with relapsing MS (RMS).

**Methods:**

In this two-year prospective study, 129 RMS patients underwent quarterly sNFL assessments and annual MRI scans. The study analyzed the correlation between individual NFL levels and past, current, and future disease activity. Group-level Z-scores were employed as a comparative measure.

**Results:**

Among the 37 participants, a total of 61 episodes of disease activity were observed. sNFL levels proved valuable in distinct ways; they were confirmatory of previous and current clinical and/or radiological activity and demonstrated a high negative predictive value for future 90 days activity. Interestingly, Z-scores marginally outperformed sNFL levels in terms of predictive accuracy, indicating the potential for alternative approaches in disease activity assessment. In our cohort, sNFL cut-offs of 10.8 pg./mL (sensitivity 27%, specificity 90%) and 14.3 pg./mL (sensitivity 15%, specificity 95%) correctly identified 7 and 4 out of 26 cases of radiological activity within 90 days, respectively, with 14 and 15% false negatives. When using lower cut-off values, individuals with sNFL levels below 5 pg/mL (with a sensitivity of 92%, specificity of 25%, and negative predictive value of 94%) were less likely to experience radiological activity within the next 3 months.

**Conclusion:**

Individual sNFL levels may potentially confirm prior or current disease activity and predict short-term future radiological activity in RMS. These findings underscore its periodic measurement as a valuable tool in RMS management and decision-making, enhancing the precision of clinical evaluation in routine practice.

## Introduction

Multiple sclerosis (MS) is a chronic inflammatory disorder of the central nervous system (CNS), predominantly affecting young adults. Neurofilament light chain (NfL), a key component of neuronal and axonal cytoskeleton proteins, is elevated not only in MS but also in other CNS diseases, serving as a general indicator of axonal damage regardless of the underlying cause (1). In MS, acute neurological impairment is assessed in clinical practice using the Expanded Disability Status Scale (EDSS) (2) and periodic magnetic resonance imaging (MRI) to detect new brain or spinal cord lesions distinguishing between active and inactive states based on gadolinium enhancement (3). The development of serum NFL (sNFL) as a longitudinal biomarker represents a significant advancement in MS monitoring (4). The minimally invasive nature of blood sample testing for sNFL and its strong correlation with CSF levels and reflect ongoing inflammatory activity (5–7), marks a breakthrough for its broader application in clinical practice. sNFL levels are influenced by factors like age and BMI, making population-based comparisons (Z-scores) adjusted for these factors a viable strategy for standardization (8, 9). However, the considerable inter-individual variability in sNFL levels poses a challenge, potentially limiting the precision of group-level assessments and underscoring the need for individualized analysis in clinical practice.

The prospective use of sNFL at an individual patient level in clinical practice remains to be established (10). This study aims to explore the clinical utility of individual sNFL in various clinical scenarios of past, current, and future disease activity in a prospective population of MS patients with quarterly measures of sNFL and annual MRI.

## Materials and methods

### Patients

In this prospective study, we enrolled 129 patients with relapsing MS (RMS) adhering to the following inclusion criteria: (a) a diagnosis of initial RMS made within the past 10 years, (b) age below 45 years at the time of inclusion, and (c) a disability status measured by EDSS of less than 4. The recruitment phase spanned from 2019 to 2021. All participants involved in the study provided written informed consent and were monitored over a two-year period with quarterly clinical evaluations and sNFL assessments, along with annual MRI scans. The study protocol was reviewed and approved by the Ethics and Scientific Committee of our center.

### Clinical definitions

The diagnosis of MS in this study was established based on the 2017 McDonald criteria (11). Regarding the definition of disease activity, we employed five distinct possibilities: (1) Any disease activity, encompassing both clinical relapse and radiological manifestations such as new gadolinium-enhancing (Gd+) lesions and/or new lesions in T2-weighted images (nT2L) and/or a clinical relapse; (2) Radiological disease activity, indicated by the presence of Gd + and/or nT2L; (3) Activity solely identified by Gd+; (4) Activity solely identified by nT2L; and (5) Clinical relapse alone. Clinical relapse was defined as an acute exacerbation of central neurological symptoms lasting over 24 h, not attributable to fever or physical stress.

Disease-modifying therapies (DMD) were prescribed based on the physician’s judgment and in accordance with local guidelines. First-line DMDs (FL-DMD) included glatiramer acetate, interferon, teriflunomide, and dimethyl fumarate. High-or moderately efficacy DMDs (HE-DMD) encompassed cladribine, fingolimod, natalizumab, anti-CD20 monoclonal antibodies, alemtuzumab, as well as a history of treatment with mitoxantrone, cyclophosphamide, and/or autologous haematopoietic stem cell transplantation (aHSCT).

### Biomarker analysis and definition of baseline sNFL

Serum samples were stored at −80°C in the biobank La Fe following the protocols for standardization in biomarker measurements (12). sNFL levels were measured as in using the NF-light Advantage SR-X kit (SIMOA, Quanterix, Lexington, MA, United States) according to the manufacturer’s instructions. All coefficients of variation were less than 20%. sNFL were measured at the first clinical assessment (baseline) and quarterly until the end of the study period. sNFL Z-scores were calculated according to the free available online tool adjusting for age^1^ (9).

### MRI

Brain and spinal cord MRI scans were conducted at baseline and annually over the two-year study period, on a 3 T Philips Archieva scanner (Philips Medical Systems, Best, Netherlands). Brain MRI protocol included an axial T1 post-Gd sequence (echo time 11 ms; repetition time 600 ms; slice thickness 3 mm) and the FLAIR sequence (transverse or sagittal planes with a thickness between 1 and 3 mm), turbo spin echo T2 sequence (3–5 mm thick), proton density sequence (3–5 mm thick). Cervical spinal cord MRI consisted of an axial 3D T1 post-gadolinium sequence (echo time 2.6 ms; repetition time 7.5 ms; slice thickness 1 mm) and axial and sagittal T2 sequences (3 mm slice thickness, both). Additional scans were performed when suspected disease activity at 6 months or sooner in cases where symptoms did not resolve.

### Statistical analysis

**Figure 1:**
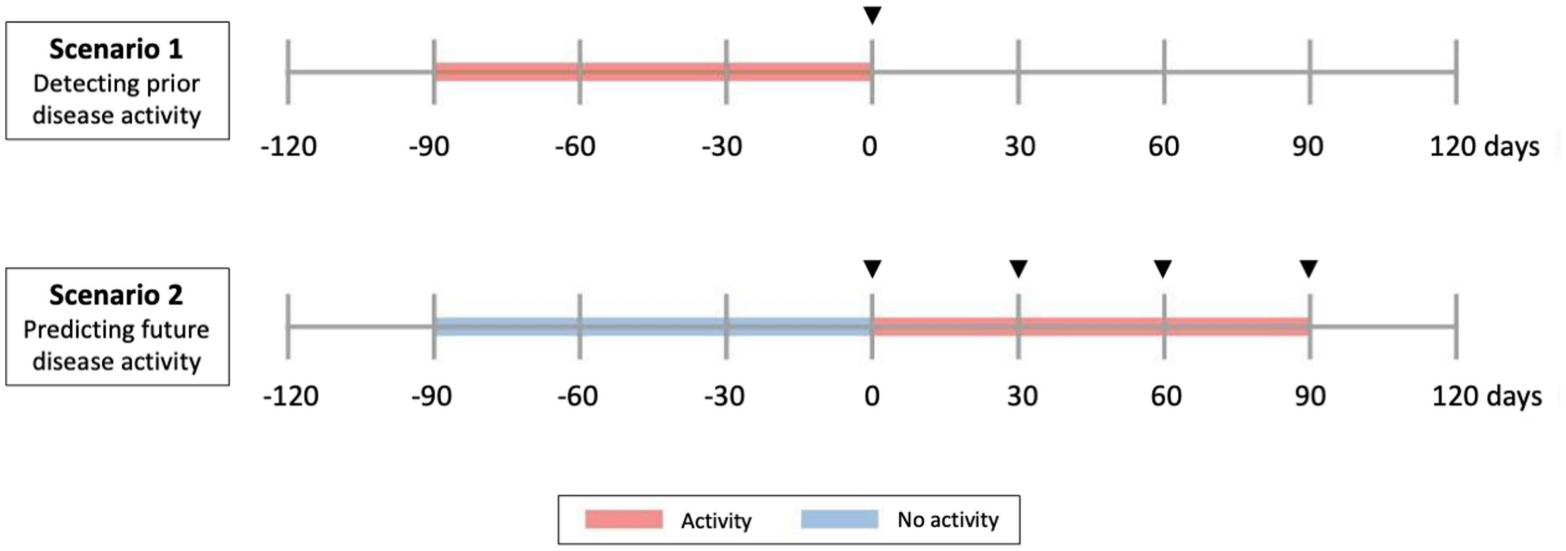
Clinical scenarios studied. Scenario 1 and 2 include all types of disease activity (radiological and clinical relapse). Scenario 1 evaluates sNfL as a predictor of current and past (previous 90 days) activity. Scenario 2 evaluates sNfL as a predictor of future (next 90 days) activity, in people without previous activity. In Scenario 1, predictor phase corresponds to a time of −90 days, while in Scenario 2 corresponds to a time of 0. Arrowheads indicate time of serum collection.

**Figure 2:**
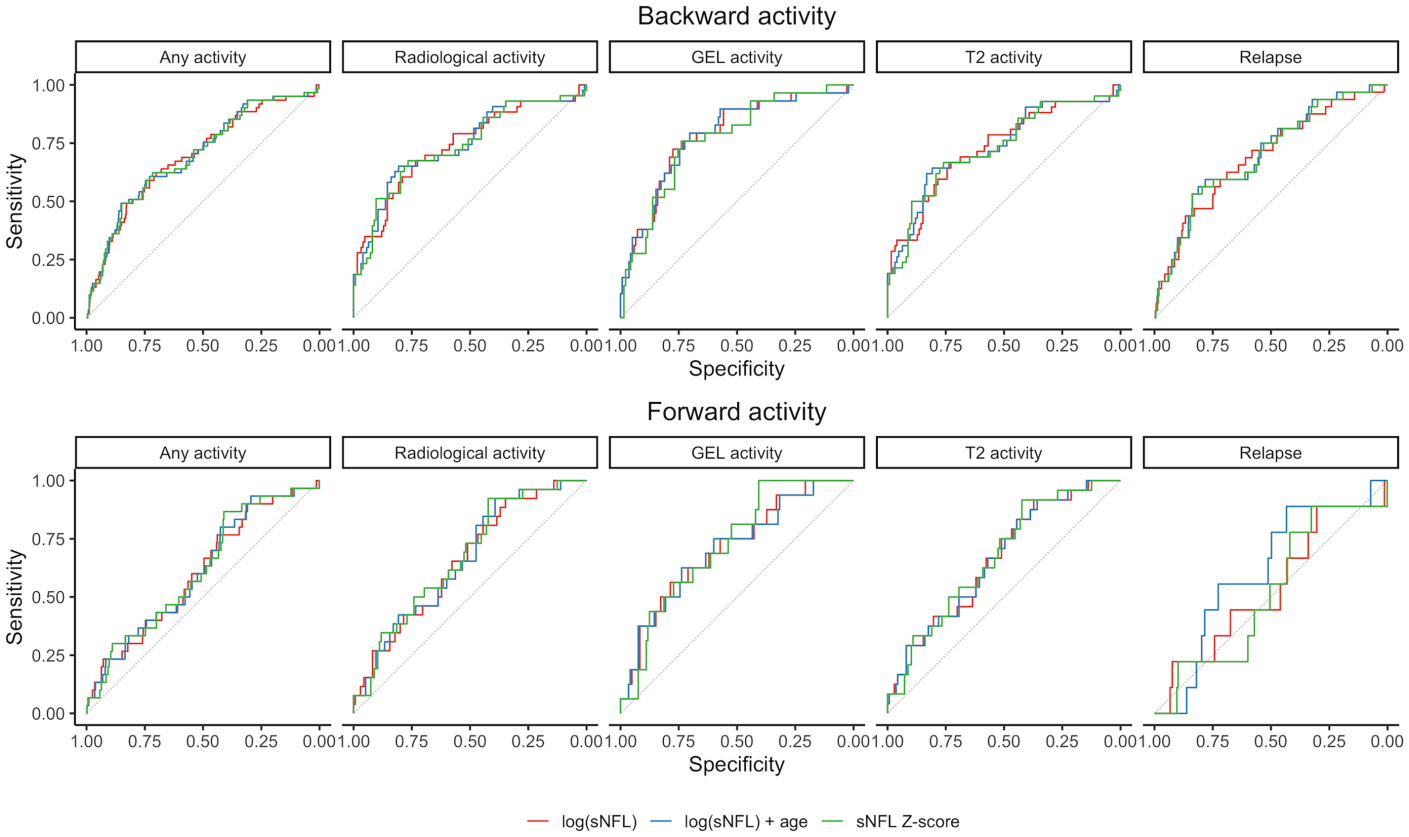
Receiver operating characteristic (ROC) curves for prediction models in the two considered scenarios. Gd+, gadolinium-enhanced lesions; sNfL, serum neurofilament light chain.

Group-level differences were analyzed using the Wilcoxon rank sum test. The relations between sNFL measures and disease activity were tested in two scenarios (Figure 1): (a) Scenario 1: Prediction of past or ongoing activity (day −90 to 0): to verify that sNFL levels react to activity; (b) Scenario 2: Prediction of “new” activity (day 1 to 90, no activity in past 90 days). For the outcomes of any disease activity and clinical relapse, we used all available visits, for radiological activity outcomes, we only included visits where an MRI was available in the associated time window. The tested predictors of disease activity of sNFL included log(sNFL), log(sNFL) + age, and sNFL Z-score. Each prediction model of disease activity was evaluated using logistic regression models and performance was assessed as area under the receiver operating characteristic curve (AUC) and *p*-values for the model predictors were computed by Chi-squared tests (Figure 2). Within each prediction task, the three models were compared by the Akaike Information Criterion (AIC).

## Results

### Patients’ demographic and clinical features

The study initially enrolled 129 patients diagnosed with RMS and among these, 128 had all quarterly sNFL measurements during the study period, making them eligible for the analyses. These patients were prospectively followed up for 2 years. The median age at the first visit was 32 years (interquartile range [IQR]: 27.6–37.9), while the median disease duration at the initial visit was 55.8 months ([IQR]: 29.5–98.5). In preparation for a more comprehensive assessment of risk before the study, we recorded sex, the presence of oligoclonal IgM bands (OCMB), the annualized relapse rate (ARR) in the year prior to study inclusion, the presence of spinal cord lesions, and calculated the modified Rio Score (mRS). The calculation of the mRS was based on the presence of radiological activity, specifically Gd + lesions and nT2L (13). These, along with other clinical and demographic characteristics, are presented in Table 1.

**Table 1:** Baseline clinical and CSF characteristics of the study cohort.

Sex female	87 (70.2)
Age	32.6 (27.6–37.9)
Disease duration (months)	56 (29.5–98.5)
CHI3L1 LP*	125.2 (83.9–179.8)
NFL LP*	680.25 (327.6–1776)
OCMB*	57 (66.3)
Myelitis	80 (65.6)
Baseline sNFL	6.90 (5.18–9.09)
mRio Score	
0	94 (76.4)
1	15 (12.2)
2	13 (10.6)
3	1 (0.8)
Baseline EDSS	1.75 (1–2.5)
Relapse previous year	28 (22.6)
Gd + enhancing lesions previous year	15 (12.1)
T2-lesions previous year	36 (29.3)
DMD	
No treatment	5 (4.0)
FL-DMD	48 (38.7)
HE-DMD	71 (57.3)

Continuous (Median (p25–p75)); Categorical (N, %); CHI3L1, chitinase-3-like protein 1; DMD, disease-modifying treatment; EDSS, expanded disability status scale; FL-DMD, First-line disease modifying treatment; Gd+, Gadolinium-enhanced; HE-DMD, High-efficacy disease modifying treatment; LP, lumbar puncture; NFL, neurofilament light-chain; OCMB, oligoclonal M bands; sNFL, serum neurofilament light-chain.

During the study period, four treatment-naïve patients initiated treatment. Additionally, nine patients escalated DMD. Eight patients had undergone aHSCT before or during the study (*n* = 3) and were categorized as being treated with high efficacy therapy, regardless of the transplantation date. Furthermore, ten patients became pregnant during the study period.

At baseline, 15 patients in the study cohort had experienced prior disease activity within the last 90 days (*N* = 29). The median sNFL levels at visits with prior disease activity were 8.98 pg./mL, ([IQR]:6.57-12.2), which were significantly higher than the median levels at visits without disease activity, which were 6.58 pg./mL, IQR: 5.00–8.54 (*p* < 0.0001). During the study, 37 patients experienced 61 events of disease activity including clinical relapses without associated radiological findings (*N* = 2), clinical relapse without associated radiological assessment (*N* = 16), clinical relapses with radiological findings (*N* = 14), and radiological findings without clinical relapse (*N* = 29) within the past 90 days. sNFL levels showed a small difference between visits with disease activity (7.19 pg./mL, IQR: 6.12–9.55) and without (6.55 pg./mL, ([IQR]:4.92-8.52)) (*p* = 0.0461).

### sNFL levels in relation to different disease activity contexts

#### Scenario 1: prediction of past or ongoing disease activity (day – 90 to 0)

Elevated sNFL levels were observed in patients with disease activity within the preceding 3 months. Specifically, increased sNFL levels were associated with any demonstrated prior disease activity including clinical relapse and/or nT2L, and/or Gd + lesions. Notably, the prediction models, including log(sNFL), log(sNFL) + age, and sNFL Z-score, were evaluated for their predictive performance. Among these, the models including sNFL Z-score emerged as the most parsimonious (lower AIC) for predicting any activity and relapse, while using log(sNFL) resulted in the most parsimonious models for the radiological activity events as detailed in Table 2. The AUCs were comparable across all models, as shown in Figure 2.

**Table 2:** Predicting the performance of sNFL measurements for disease activity in the past 90 days (from day −90 to day 0).

sNFL measure	Any activity (radiographic or relapse) 51/726 events in 34/124 subjects	Radiographic activity (GEL/T2) 40/726 events in 27/124 subjects	GEL activity 22/726 events in 17/124 subjects	T2 activity 39/726 events in 27/124 subjects	Relapse 24/726 events in 19/124 subjects
log(sNFL)	AIC = 352.90	AIC = 303.52	AIC = 196.18	AIC = 298.68	AIC = 199.66
	AUC = 0.72	AUC = 0.68	AUC = 0.69	AUC = 0.68	AUC = 0.77
	*p* < 0.0001	*p* = 0.0015	*p* = 0.0254	*p* = 0.0023	*p* = 0.0001
log(sNFL) + age	AIC = 351.91	AIC = 300.79	AIC = 195.96	AIC = 296.75	AIC = 199.27
	AUC = 0.72	AUC = 0.67	AUC = 0.69	AUC = 0.67	AUC = 0.78
	*p* < 0.0001	*p* = 0.0006	*p* = 0.0272	*p* = 0.0014	*p* = 0.0002
sNFL Z-score	AIC = 345.75	AIC = 297.26	AIC = 191.41	AIC = 293.22	AIC = 193.00
	AUC = 0.72	AUC = 0.69	AUC = 0.70	AUC = 0.69	AUC = 0.77
	*p* < 0.0001	*p* = 0.0001	*p* = 0.0018	*p* = 0.0001	*p* < 0.0001

#### Scenario 2: prediction of “new” disease activity (day 1 to 90, without activity in the past 90 days)

In scenarios where no disease activity was observed in the previous 3 months, sNFL levels and the associated Z-scores exhibited predictive potential for future radiological activity within the subsequent 3 months (from day 1 to 90) although with modest AUCs ranging between 0.66 and 0.73. It is noteworthy that there was no evidence to suggest that sNFL levels could predict future clinical relapses. The sNFL Z-score model showed marginally better performance than the log(sNFL) model. Elevated sNFL levels more accurately predicted the emergence of new Gd + in MRI than the appearance of new T2L or a combination of clinical and radiological activity, as presented in Table 3.

**Table 3:** Predicting the performance of sNFL measurements for disease activity in the upcoming 90 days (day 1 to 90, no prior activity).

sNFL measure	Any activity (radiographic or relapse) 31/675 events in 26/124 subjects	Radiographic activity (GEL/T2) 27/675 events in 24/124 subjects	GEL activity 13/675 events in 11/124 subjects	T2 activity 24/675 events in 22/124 subjects	Relapse 8/675 events in 6/124 subjects
log(sNFL)	AIC = 246.13	AIC = 219.38	AIC = 125.54	AIC = 202.76	AIC = 90.39
	AUC = 0.64	AUC = 0.66	AUC = 0.67	AUC = 0.64	AUC = 0.58
	*p* = 0.0021	*p* = 0.0008	*p* = 0.0086	*p* = 0.0035	*p* = 0.4886
log(sNFL) + age	AIC = 247.33	AIC = 220.40	AIC = 127.16	AIC = 203.64	AIC = 92.18
	AUC = 0.65	AUC = 0.68	AUC = 0.69	AUC = 0.66	AUC = 0.58
	*p* = 0.0060	*p* = 0.0021	*p* = 0.0262	*p* = 0.0080	*p* = 0.7072
sNFL Z-score	AIC = 244.52	AIC = 217.32	AIC = 124.59	AIC = 201.71	AIC = 90.18
	AUC = 0.66	AUC = 0.69	AUC = 0.71	AUC = 0.66	AUC = 0.58
	*p* = 0.0009	*p* = 0.0003	*p* = 0.0051	*p* = 0.0020	*p* = 0.4050

### Suggested sNFL levels for guiding clinical decision-making in disease activity prediction

The determination of an appropriate cut-off value for sNFL holds significance in clinical decision-making, particularly in the context of diagnosing relapse and guiding early treatment modifications, especially when immediate access to MRI is unavailable. In our present cohort, we conducted a comprehensive analysis of various sNFL cut-off values to assess their predictive utility for disease activity.

Our findings revealed that a cut-off value of 10.8 pg./mL for sNFL exhibited a sensitivity of 27% and a specificity of 90% in predicting future radiological activity. This implies that sNFL levels at or above 10.8 pg./mL were associated with a heightened likelihood of forthcoming disease activity, facilitating timely intervention. However, it is important to note that this threshold also resulted in 14 false positive predictions out of 26 events, highlighting the need for cautious interpretation when values approach this threshold. Nonetheless, the negative predictive value (NPV) for this cut-off value was 86%, indicating that 14% of individuals with sNFL levels below 10.8 pg./mL would still exhibit radiological activity within the subsequent 3 months.

In contrast, a higher cut-off value of 14.3 pg./mL demonstrated a sensitivity of 15% and a specificity of 95%. This threshold accurately identified 4 out of 26 events of radiological activity, albeit with 7 false positives. The NPV for this cut-off was 85%, suggesting that 15% of individuals with sNFL levels below 14.3 pg./mL might still experience radiological activity within the next 3 months.

Further exploration of a lower cut-off value of 5 pg./mL unveiled a high sensitivity of 92% but a lower specificity of 25%. The NPV for this threshold was 94%, signifying that only 6% of individuals with sNFL levels below 5 pg./mL would encounter radiological activity in the next 3 months.

## Discussion

Assessing active inflammation in RMS, particularly when clinical manifestations are subtle, remains a significant challenge in routine clinical practice. Our study sought to evaluate the utility of individual sNFL levels compared with population-based Z-scores in various clinical contexts, aiming to assist clinicians in decision-making regarding disease activity. This investigation was conducted within a well-controlled cohort over a two-year period.

sNFL is a surrogate marker of neuroaxonal damage with a robust correlation with CSF NFL levels. To this regard, although some studies have reported weak correlations between both measures, a systematic review and meta-analysis (7) found a strong pooled correlation coefficient of r = 0.72, confirmed in our cohort. In a comprehensive evaluation of the utility of longitudinal sNFL assessment, we considered two clinical scenarios to evaluate its short-term predictive value. sNFL levels and associated Z-scores were predictive of disease activity in the past 3 months (AUCs 0.69–0.78) and prospectively assessed radiological activity (AUCs 0.66–0.73). These findings are consistent with other studies that found a robust association of sNFL with radiological outcomes, potentially due to concurrent asymptomatic MRI activity at the time of serum collection (5, 14–16).

Longitudinal tracking of sNFL levels may provide a more comprehensive understanding of disease progression than relying solely on baseline measurements (10, 14, 17, 18). However, the lack of standardized cut-off values poses a significant challenge for their use in clinical practice. The selection of a clinical cut-off should strike a balance between achieving the desired sensitivity and specificity. In Thebault’s prospective cohort study, both baseline sNFL levels and subsequent elevations during follow-up were predictive of relapses, particularly in individuals with baseline sNFL levels below 10 pg./mL, demonstrating that a two-fold increase during follow-up corresponded to a 41% increased risk of experiencing a relapse. Ziemssen and colleagues (19) established a cut-off of 9.3 pg./mL to differentiate high and low sNFL levels in their MS population. In an RMS cohort, Kuhle and colleagues (6) identified a cut-off of 30 pg./mL to distinguish between low and medium sNFL levels, and 60 pg./mL to separate medium from high levels, with the threshold for healthy controls being around 16.5 pg./mL. The variability in reported cut-off levels can be attributed to factors such as methodology or differences in the target population. Based on the results within our cohort and with the aim of identifying a value that could guide therapeutic interventions, it is advantageous to minimize false positives. In this context, a higher cut-off value like 14.3 pg./mL, offering a high specificity may be favored. However, this choice may come at the expense of reduced sensitivity, potentially resulting in the oversight of cases exhibiting asymptomatic disease activity. Conversely, considering a lower cut-off value such as 5 pg./mL becomes beneficial for detecting nearly all instances of disease activity, thanks to its high sensitivity. However, this heightened sensitivity is accompanied by lower specificity, leading to a higher likelihood of false positives. The mid-range cut-off value of 10.8 pg./mL strikes a balance between sensitivity and specificity, presenting a reasonable compromise. However, in situations where disease activity is not anticipated due to a patient’s clinical stability and on those treated with high-efficacy treatments, sNFL values lower than 5 pg./mL may provide reassurance that ongoing disease activity is unlikely due to its high NPV. This idea has been explored by Uher et al., who found that sNFL levels below the 30th percentile had a very low probability of radiologic disease activity during the preceding year, suggesting the possibility of substituting annual brain MRI monitoring in clinically stable patients (20). In recent studies, low sNFL levels have been shown to effectively exclude clinical or subclinical disease activity, while even minor increases may indicate disease progression during relapse-free periods (19, 21).

The frequency of sNFL level assessment in MS is a topic of ongoing research, and the specific recommended frequency for its clinical management in MS is still evolving. Concerning the dynamics of sNFL, it is important to consider its half-life, which plays a pivotal role in determining the optimal frequency for monitoring disease activity in MS patients. Bergman et al. showed that after CNS injury, both CSFNFL and sNFL returned to baseline levels after 6–9 months after elevation (22). In another investigation focused on MS patients sampled at the time of relapse, sNFL showed an increase in the 5 months prior to reaching its peak at clinical relapse, followed by recovery to previous levels within 4–5 months (17). Furthermore, a separate observational cohort found that sNFL levels were elevated by one third within a 3 months window around Gd + lesions compared to samples taken during remission (23). Some authors advocate for quarterly measures as a valuable approach when monitoring disease activity in MS (24). From our perspective, sNFL demonstrate a remarkable ability to confirm prior activity (clinical and/or radiological), regardless of whether patients experience relapses. Therefore, we recommend quarterly measurements whenever possible. In cases where quarterly measurements are not available, they should be conducted whenever there is suspicion of a relapse or when disease activity appears uncontrolled. Elevated sNFL levels could potentially serve as a substitute for MRI in cases where fast therapeutic decisions need to be made for symptomatic patients with elevated sNFL, but this possibility warrants future studies.

Inter-individual sNFL differences may be due to distinct degrees of inflammatory activity and neurodegeneration, and other variables like age as the strongest predictor factor (9, 25), BMI (8, 9), renal function (26, 27), and other comorbidities. To circumvent the limitations, our study compared individual sNFL levels with age- and BMI-matched population-based Z-scores (9). Both approaches yielded comparable results, with sNFL Z-scores slightly favoring confirmation of prior or current disease activity, suggesting that individual levels may be also a proper and more direct approach.

Our study had certain limitations, including a relatively modest sample size and a two-year follow-up period, which may limit our ability to assess long-term prognosis. Additionally, we did not adjust our results for factors such as BMI or renal function. However, it is noteworthy that these variables were homogeneous within a relatively young study cohort. Specifically, there were no cases of renal impairment. Furthermore, research in other diseases, including those affecting the elderly, suggests that adjusting for BMI and renal function might not significantly alter the predictive role of sNFL in disease course (28). We acknowledge that our investigation did not assess the prognostic value of baseline sNFL levels in the absence of relapse to predict disease progression. This aspect is crucial and aligns with the findings of prior studies, which have linked elevated baseline sNFL levels to poorer long-term MRI outcomes, increased EDSS scores, and MRI atrophy over extended follow-up periods (29–31). These studies have provided valuable insights into the potential clinical relevance of baseline sNFL levels, highlighting their association with worse long-term MRI outcomes and disease progression. Yet, it is important to note that the cross-sectional nature of baseline sNFL measurements primarily categorizes patients into risk groups and does not fully elucidate the role of NfL in monitoring RMS patients, especially in a disease characterized by fluctuating activity and remission phases, along with a biomarker exhibiting a highly variable longitudinal profile (14, 25).

In conclusion, our study suggests that longitudinal sNFL levels and derived Z-scores may be valuable tools in confirming previous or concurrent disease activity in RMS. Their most significant utility in daily practice, however, may lie in ruling out future short-term radiological activity. The insights gained from our study, along with those from other research, could inform future decisions in patient management.

## Data availability statement

The raw data supporting the conclusions of this article will be made available by the authors, without undue reservation.

## Ethics statement

The studies involving humans were approved by IIS La Fe Ethics and Scientific Committee (PT17/0015/0043). The studies were conducted in accordance with the local legislation and institutional requirements. The participants provided their written informed consent to participate in this study.

## Author contributions

LS-T: Conceptualization, Data curation, Investigation, Methodology, Project administration, Visualization, Writing – original draft, Writing – review & editing. LR: Data curation, Formal analysis, Methodology, Software, Validation, Writing – original draft, Writing – review & editing. JC-M: Data curation, Investigation, Writing – review & editing. SR: Data curation, Investigation, Writing – review & editing. SG-P: Conceptualization, Data curation, Funding acquisition, Investigation, Methodology, Resources, Software, Supervision, Validation, Writing – original draft, Writing – review & editing.

## Footnotes

^1^https://shiny.dkfbasel.ch/baselnflreference/
